# Changes in toothbrushing and factors associated with less than twice daily toothbrushing by the age of four in the FinnBrain Birth Cohort Study

**DOI:** 10.2340/aos.v85.46661

**Published:** 2026-08-11

**Authors:** Hanna Suokko, Mimmi Tolvanen, Jorma Virtanen, Auli Suominen, Linnea Karlsson, Hasse Karlsson, Satu Lahti

**Affiliations:** a Department of Community Dentistry, University of Turku, Turku, Finland; b Unit of Medical Education, Education and Research, The Wellbeing Services County of Pirkanmaa, Tampere, Finland; c Emergency Services Academy Finland, Kuopio, Finland; d Faculty of Medicine, University of Bergen, Bergen, Norway; e FinnBrain Birth Cohort Study, Department of Clinical Medicine, Turku Brain and Mind Center, University of Turku, Turku, Finland; f Centre for Population Health Research, University of Turku and Wellbeing Services County of Southwest Finland, Turku, Finland; g Unit of Public Health, Department of Clinical Medicine, University of Turku and Turku University Hospital, Turku, Finland; h Department of Psychiatry, Unit of Child Psychiatry, Turku University Hospital, Turku, Finland; i Department of Psychiatry, University of Turku and Turku University Hospital, Turku, Finland

**Keywords:** Oral health behavior, toothbrushing, children, parents

## Abstract

This study examined toothbrushing and its associated factors in children aged 1–4 years, using data from 306 mother-father-dyads and their 308 children participating in the FinnBrain Birth Cohort Study. Longitudinal multivariable regression analysis was conducted using the generalized estimating equation method to model the outcome, parent-reported brushing of a child’s teeth less than twice daily at the child’s age of 12, 24 and 48 months, controlling for the intraindividual correlation between repeated measurements using compound symmetry (correlation structure). The independent variables as time-varying covariates from 12 to 48 months were mother’s and father’s own toothbrushing, couple relationship satisfaction, number of positive life events of father, and fixed covariates were number of siblings, education level of mother and age of parents. Among the children, 50% had stable good and 16% stable poor toothbrushing habits from 1 to 4 years. Both parents’ less than twice daily brushing was strongly associated with less than twice daily brushing of a child’s teeth. The strength of the association between brushing mother’s own and the child’s teeth varied across time, whereas this association in fathers remained consistent. Early family-based intervention is crucial for promoting parents’ own brushing habits and thereby supporting children’s oral hygiene.

## Introduction

Young children acquire their toothbrushing habits mostly from their parents, as they are their primary social environment [1–4]. These habits appear to become established before the age of 5 years and remain relatively stable over time [5–7].

Previous studies have shown that mother’s own toothbrushing frequency is strongly associated with child’s brushing frequency, but few studies have examined the role of fathers [8–16]. In our previous studies, we found that both mothers’ and fathers’ toothbrushing frequency were strongly associated with the brushing frequency of their child, both cross-sectionally and longitudinally. Moreover, poor toothbrushing frequency tended to persist over time among both parents and children [15, 16].

In addition to parents acting as a model, family demographics, such as education, are associated with parents’ own toothbrushing, which, in turn, shapes their child’s toothbrushing. Lower levels of education are generally associated with lower toothbrushing frequency [17–21]. However, among preschool-aged children, a similar consistent association has not been demonstrated [6, 15, 16, 22]. Furthermore, some findings suggest that families with several children may have fewer resources for daily toothbrushing routines, potentially reducing the frequency of toothbrushing in children [15, 23]. In addition, some studies have indicated that older parents reported to brush their child’s teeth more frequently than younger parents [15, 23, 24].

Parents’ overall well-being plays an important role in shaping health behaviors within the family. Factors such as significant life events and couple relationship satisfaction may change the stability of family life and daily caregiving behaviors [25, 26]. They may disrupt established routines and reduce the consistency of everyday practices, including maintaining young children’s oral hygiene. Moreover, anxiety and depression commonly occur during the postpartum period and may persist for several years following childbirth [27]. Regardless of the source of daily challenges or mental health problems within family life, these issues may negatively affect parents’ overall well-being and alter health behaviors [25–33]. They may also be related to how parents care for their young children’s oral hygiene [23].

Parents’ well-being has been shown to be associated with their own toothbrushing [34–40]. Maternal depression and anxiety have been found to negatively impact mother’s toothbrushing frequency [38–40]. These findings for toothbrushing were more prominent in those with high levels of depression and anxiety. Furthermore, both men and women with a high number of depressive or anxiety symptoms report lower toothbrushing frequency than those with no or only a few depressive or anxiety symptoms [38]. On the other hand, one study demonstrated an association between depression and decreased toothbrushing frequency, but not with anxiety and toothbrushing frequency, whereas another found anxiety to be the key factor, when both conditions were examined within the same study [39, 40]. Moreover, findings on the association between parent’s stress and toothbrushing frequency of their children or early childhood caries are inconsistent. For example, a study by Litt et al. [41] indicated that parents’ stressful life events had an inverse relationship with caries prevalence of their children. In contrast, some studies report no significant association or even positive association between parent’s stress and early childhood caries [10, 42–45]. These findings are based on cross-sectional studies. To the best of our knowledge, the association between changes in parents’ well-being and changes in brushing of their child’s teeth has not yet been studied.

The aim of this study was to examine toothbrushing of a child from 1 to 4 years old. Our specific aim was to find family-related factors associated with changes and stability in brushing a child’s teeth during this period. Special focus was on identifying factors associated with less than twice daily toothbrushing. In addition, we aimed to assess whether parents reported the frequency of brushing their child’s teeth consistently.

## Material and methods

This study is part of the multidisciplinary FinnBrain Birth Cohort Study (www.finnbrain.fi) in Finland and a secondary analysis of longitudinal data. The FinnBrain Birth Cohort Study examines combined influences of environment and genes on child’s brain development and health [46]. The participants were recruited after free-of-charge ultrasound scans by municipal maternity clinics during the first trimester of their pregnancy (gestational week [gwk] 12) in the South-Western Hospital District and the Åland Islands in Finland from 2011 to 2015. Mothers were asked to invite their partners, who had not attended the ultrasound appointment, to participate in the study. The coverage of pregnant women attending ultrasonography appointments was close to 100% in the population of the region.

**Figure 1:**
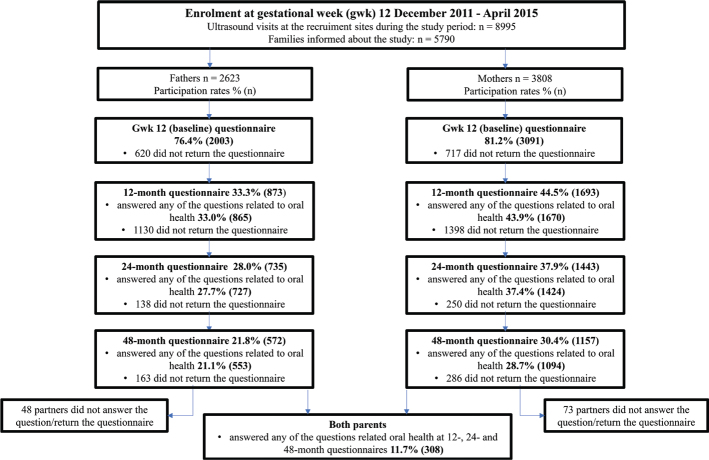
Flow chart presenting the number of participants at different phases of the study.

A total of 66% (3808/5790) of pregnant women who attended ultrasound scans at gwk 12, and 2623 fathers/partners decided to participate in the study. The Ethics Committee of the Hospital District of Southwest Finland has approved the study protocol (14 June 2011, ETMK: 57/180/2011 § 168). Figure 1 presents the number of participants at different phases of the study. The parents gave written informed consent on their own and on their child’s behalf before participating.

Both parents were required to have answered at least one question related to their own or their child’s toothbrushing at the 12‑, 24‑, or 48‑month time points to be included in the analysis. In total, 306 mother-father pairs, representing 18.4% of mothers and 35.5% of fathers who answered any toothbrushing-related questions at the 12‑month time point, and their 308 children were included in the analysis. Attrition analysis of overall loss in the FinnBrain Cohort study has been presented earlier [46]; those who have dropped out of the study were statistically significantly younger and had lower education and were more often male [26].

At the 12- and 24-month time points, the frequency of toothbrushing was asked with a question with seven response alternatives: 3–4 times per day, twice daily, once daily, 2–3 times per week, once a week, twice a month and seldom or never. At the 48-month time point, the question on the frequency of toothbrushing was asked with an open question: How many times do you and your child brush/es his/her teeth a day? All toothbrushing frequencies were dichotomized as good (twice daily or more often) and poor (less than twice daily). Dichotomizations were done according to Finnish Current Care Guidelines [47].

The selection of the family‑related variables used in the analysis, including family demographics and well‑being indicators, is justified in the introduction. Child’s sex assigned at birth, age and educational level of the mother and father were inquired at the baseline (gwk 12). Data on the number of siblings in the household were collected at the time points of 12, 24 and 48 months with an open question: Are there any siblings in the family and when was the sibling born? The variable of siblings was dichotomized as follows: yes = 0 (the child has siblings), no = 1 (the child has no siblings). The parents’ age was used as continuous variable. Education was chosen from different socio-economic variables due to its best predictive ability in this population [48]. Education was categorized into two levels: low (high school/vocational ≤ 12 years) = 1, medium (polytechnic) and high (university degree or comparable) = 0. The categorization is based on both the number of years and the orientation of education according to the Finnish system, which has compulsory level (9 years), secondary level with vocational or general/academic (11–12 years) orientation, and further level with vocational (polytechnic) or academic (university degree or comparable) orientation.

Family well-being variables examined in this study included couple relationship satisfaction, life events occurring within the family during the previous year, and parents’ depression and anxiety symptoms. Data on life events and couple relationship satisfaction were collected through questionnaires administered at the child’s age of 12, 24, and 48 months. Information about parents’ depression and anxiety symptoms was collected at 24 and 48 months postpartum.

Couple relationship satisfaction was measured by a valid and reliable scale, namely Revised Dyadic Adjustment Scale (RDAS) [49]. This questionnaire was administered at the children’s ages of 24 and 48 months. RDAS consists of 14 items, each scaled from 1 to 6. Thus, the minimum score for RDAS is 14 and the maximum score is 84. Lower scores indicate greater relationship satisfaction, while higher scores indicate poorer relationship satisfaction. The sum scores of the RDAS were used as continuous variable in statistical modeling.

The questionnaire on life events included 17 events (for example: mother’s return to working life, child starting daycare, parents’ divorce, unemployment, death of a grandparent, moving into a new apartment). Events were assessed by a questionnaire with a 5-point scale on each item, of which 1 or 2 indicated a perceived positive life event and 4 or 5 indicated a perceived negative life event. Life events were summed into two different variables, number of positive and number of negative life events. Positive and negative life event variables were further classified into two categories: no life events = 0 (no), one or more life events = 1 (yes).

Depressive symptoms were measured using Edinburgh Postnatal Depression Scale (EPDS). The EPDS is a widely studied questionnaire and a validated measure of depressive symptoms during both prenatal and postnatal period in both mothers and fathers. [50–54]. It consists of 10 items that are scored on a 4-point Likert scale (from 0 to 3), the sum score ranging between 0 and 30, with higher scores indicating more depressive symptoms. The sum scores of the EPDS were used as a continuous variable in statistical modeling.

Anxiety symptoms were assessed using the anxiety subscale of Symptom Checklist 90 (SCL-90^ANX^) [55]. The Finnish version of the SCL has shown to be a valid and reliable measure of anxiety symptoms [56]. The anxiety subscale of SCL-90 consists of 10 items scored on a 5-point Likert scale (from 0 to 4), and the range of total sum scores is 0–40, with higher scores indicating more anxiety symptoms. The sum scores of SCL-90^ANX^ were used as a continuous variable in statistical modeling.

All analyses were conducted separately for mothers and fathers. Bivariate associations between categorical family-related variables (education of a parent, number of siblings, sex of a child, life events and RDAS) and whether the mother/father reported brushing of a child’s teeth at least twice daily, were assessed using cross-tabulations and Pearson chi-squared tests at each time point separately (at 12 months, 24 months and 48 months). The associations between continuous family-related variables (parent’s age, RDAS, EPDS, SCL-90^ANX^) and the variable of mother/father reporting brushing of a child’s teeth twice daily, were analyzed using nonparametric Mann Whitney U test, due to asymmetry of distribution. The Wilcoxon signed-rank test and Cohen’s kappa test were used when examining whether mothers reported the frequency of brushing their child’s teeth differently from fathers.

The generalized estimating equations (GEE) for logistic regression models were used to analyze the longitudinal associations between parent-reported brushing of a child’s teeth (ref. at least twice daily) and each family-related variable over 12, 24 and 48 months. The Wald test was used to assess the associations between the constant variables (parent’s age and educational level and child’s sex) and parent-reported brushing of a child’s teeth. The final multivariable longitudinal regression analysis was conducted applying GEE-method for modeling the outcome, parent-reported brushing of a child’s teeth (ref. at least twice daily) throughout the study, controlling for the intraindividual correlation between repeated measurements using compound symmetry (CS correlation structure). The independent variables as time-varying covariates from 12 to 48 months were mother’s and father’s own toothbrushing, couple relationship satisfaction, positive life events of father, and as fixed covariates were siblings, education level and age of parent. The coding of these variables is shown in Table 1. We also included interaction terms between time and all other independent variables. Interaction terms were eliminated if they failed to meet the significance threshold (*p* < 0.05) or did not enhance model performance. Statistical analyses were conducted using SPSS statistics for Windows (IBM), version 26.0, or the statistical analysis software SAS version 9.4 (SAS Institute).

**Table 1:** Proportion (%) or means and standard deviations (SD) of family-related variables and toothbrushing, presented separately for mothers and fathers at child’s age of 12, 24 and 48 months in the FinnBrain Birth Cohort study (*n* = 308).

Variable	Categories	Code	Mother				Father			
			12 mos	24 mos	48 mos	*p*	12 mos	24 mos	48 mos	*p*
Toothbrushing % (child)	At least twice daily	0	58.3	75.3	76.8	< 0.001^a^	57.0	71.4	77.6	0.134^a^
	Less than twice daily	1	41.7	26.5	23.2		43.0	28.6	22.4	
Toothbrushing % (own)	At least twice daily	0	81.1	84.1	82.1	< 0.001^a^	73.6	72.4	75.0	< 0.001^a^
	Less than twice daily	1	18.9	15.9	17.9		26.4	27.6	25.0	
Education %	Low	1	17.6			0.134^b^	31.6			0.127^b^
	Medium/High	0	82.4				68.4			
Age mean (SD)		-	32.5(4.1)			0.640^b^	34.4(4.9)			0.277^b^
Sex of a child %	Girl	0	48.1			0.779^b^	48.1			0.855^b^
	Boy	1	51.9				51.9			
Siblings %	Yes	0	35.9	49.2	73.7	0.551^a^	37.1	50.0	72.9	0.585^a^
	No	1	64.1	51.8	26.3		62.9	50.0	27.1	
Life events %	Positive ≥ 1	1	25.4	32.1	27.0	0.257^a^	15.0	28.6	20.7	0.070^a^
	Positive = 0	0	74.6	67.9	73.0		85.0	71.4	79.3	
	Negative ≥ 1	-	23.5	25.3	25.8	0.308^a^	17.9	18.5	15.2	0.827^a^
	Negative = 0	-	76.5	74.7	74.2		82.1	81.5	84.8	
RDAS mean (SD)		-	31.8(6.2)	31.1(6.6)	31.1(6.7)	0.836^a^	31.9(6.7)	32.1(7.7)	31.1(7.0)	0.085^a^
EPDS mean (SD)		-		4.5 (4.2)	4.8 (4.6)	0.199^a^		3.4 (3.7)	3.7 (4.1)	0.505^a^
SCL-90^ANX^ mean (SD)		-		2.9 (4.4)	3.4 (4.5)	0.051^a^		2.1 (3.5)	2.5 (3.5)	0.379^a^

Note: The coding used in the final multivariable longitudinal regression analysis is also given.

Mos: months; RDAS: Revised Dyadic Adjustment Scale; EPDS: Edinburgh Postnatal Depression Scale; SCL-90^ANX^: Symptom Checklist 90 Anxiety Subscale.

a *P*-values for Generalized estimating equations (GEE) for the longitudinal associations between parent-reported brushing of a child’s teeth (ref. at least twice daily) and family-related variables.

b *P*-values for associations between parent-reported brushing of a child’s teeth (ref. at least twice daily) and constant family-related variables over time using Wald test.

## Results

The average age of mothers at childbirth was 32.5 years (SD 4.1), and the average age of the fathers at childbirth was 34.4 years (SD 4.9). The toothbrushing and family-related variables at the child’s age of 12, 24 and 48 months are shown in Table 1. The majority of families had no significant life events during measurement period. About three-quarters of children had either younger or older siblings at the age of 48 months. The number of girls among the children was slightly lower than that of boys. The prevalence of brushing of a child’s teeth reported by mother increased from 1 to 4 years of age, but similar change was not observed in the father-reported brushing of a child’s teeth. According to GEE, the associations between parent-reported brushing of a child’s teeth and family-related variables were not statistically significant over 12, 24, and 48 months.

**Figure 2:**
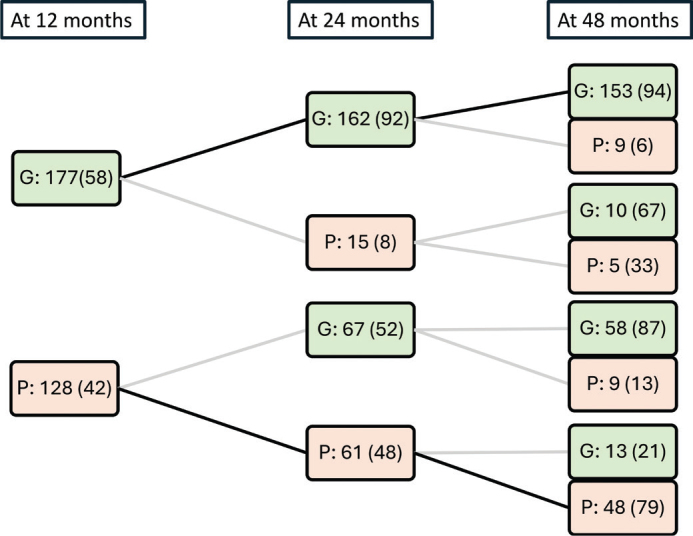
Stage transitions for mother-reported brushing of a child’s teeth from 12 to 48 months of age presented as *n* (%) among children in the FinnBrain Birth Cohort Study (*n* = 305). Good behavior (G): brushing of a child’s teeth at least twice daily, poor behavior (*P*): brushing of a child’s teeth less than twice daily.

Figure 2 reports the changes in mother-reported brushing of a child’s teeth from 12 to 48 months of age. Half (50%) of the children had stable good toothbrushing from age 1 to 4 years, while about one-sixth of the children had stable poor toothbrushing throughout this period. However, toothbrushing frequency varied with one-third of the children. Within the varying toothbrushing group, two-thirds improved their brushing frequency, and two tenths impaired their brushing frequency. Rest of the children’s toothbrushing was fluctuating, from good to poor and poor to good, throughout the measurement period.

Associations between categorical family-related variables and the mother- and father-reported brushing of a child’s teeth at least twice daily are presented in Tables 2 and 3, respectively. In mothers, only one statistically significant association was observed. Those mothers who had experienced at least one positive life event were more likely to report brushing their child’s teeth less than twice daily than did mothers who had not experienced any positive life events at 12 months. In fathers, two associations were found. Younger fathers were more likely to brush their child’s teeth at least twice daily than older fathers at 12 months. When the child was 2 years old, fathers with a lower level of education were more likely to brush their child’s teeth less often than fathers with higher levels of education did.

**Table 2:** Mother-reported brushing of a child’s teeth at least twice daily at the age of 12, 24 and 48 months according to family-related variables in the FinnBrain Birth Cohort (*n* = 305).

Grouping variables		Mother-reported brushing of a child’s teeth at least twice daily		
		12 months	24 months	48 months
		*n* (%)	*n* (%)	*n* (%)
Education	Low	26 (50.0)	37 (71.2)	37 (71.2)
	Medium/High	147 (60.7)	188 (77.4)	189 (78.4)
Siblings	Yes	60 (56.1)	114 (77.6)	172 (76.4)
	No	116 (61.1)	111 (73.0)	63 (77.8)
Sex of a child	Girl	86 (58.5)	112 (75.7)	116 (78.4)
	Boy	93 (58.1)	120 (75.0)	119 (75.3)
Positive life events	No	142 (62.0)^†^	160 (76.6)	135 (79.9)
	Yes	37 (47.4)	72 (72.7)	47 (75.8)
Negative life events^‡^	No	137 (58.3)	169 (73.5)	136 (79.5)
	Yes	42 (58.3)	63 (80.8)	46 (76.7)

Continuous variables		mean (SD)	mean (SD)	mean (SD)

Maternal age	At least twice daily	31.2 (4.1)	31.4 (4.1)	31.7 (4.0)
	Less than twice daily	31.9 (4.2)	32.0 (4.2)	31.2 (4.7)
RDAS^‡^	At least twice daily	31.8 (6.2)	30.7 (6.5)	31.2 (6.7)
	Less than twice daily	31.7 (6.2)	32.2 (6.8)	31.1 (6.8)
EPDS	At least twice daily		4.2 (4.0)	4.7( 4.6)
	Less than twice daily		5.3 (4.6)	5.3 (4.4)
SCL-90^ANX^	At least twice daily		2.7 (4.2)	3.1 (4.0)
	Less than twice daily		3.5 (4.9)	4.4 (5.7)

RDAS, Revised Dyadic Adjustment Scale; EPDS, Edinburgh Postnatal Depression Scale; SCL-90^ANX^, Symptom Checklist 90 Anxiety Subscale.

† *P* < 0.05 for chi-squared tests separately for each time point and within the groups of mothers.

# *P* < 0.05 for Mann Whitney U test within the groups of mothers.

‡ Missing values: negative life events at 48 months: *n* = 75, RDAS at 48 months: *n* = 91. Others have less than 15 missing values.

**Table 3:** Father-reported brushing of a child’s teeth at least twice daily at the age of 12, 24 and 48 months according to family-related variables in the FinnBrain Birth Cohort (*n* = 307).

Grouping variables		Father-reported brushing of a child’s teeth at least twice daily		
		12 months	24 months	48 months
		*n* (%)	*n* (%)	*n* (%)
Education	Low	51 (56.0)	58 (63.0)^†^	66 (71.7)
	Medium/High	117 (58.8)	149 (74.9)	160 (80.4)
Siblings	Yes	56 (52.8)	106 (72.1)	179 (78.9)
	No	115 (60.2)	152 (70.4)	60 (74.1)
Sex of a child	Girl	82 (55.4)	108 (73.0)	118 (79.7)
	Boy	93 (58.5)	112 (70.0)	121 (75.6)
Positive life events^‡^	No	153 (58.6)	152 (69.1)	132 (76.7)
	Yes	22 (47.8)	68 (77.3)	38 (84.4)
Negative life events^‡^	No	146 (57.9)	179 (71.3)	141 (76.6)
	Yes	29 (52.7)	41 (71.9)	29 (87.9)

Continuous variables		mean (SD)	mean (SD)	mean (SD)

Paternal age	At least twice daily	32.8 (4.8)^#^	33.4 (4.8)	33.5 (4.8)
	Less than twice daily	34.3 (5.0)	33.6 (5.4)	33.2 (5.3)
RDAS^‡^	At least twice daily	31.7 (6.5)	30.8 (6.4)	31.9 (7.5)
	Less than twice daily	32.2 (6.9)	31.8 (8.5)	32.6 (8.4)
EPDS	At least twice daily		3.4 (3.8)	3.6 (4.1)
	Less than twice daily		3.5 (3.2)	4.2 (4.1)
SCL-90^ANX^	At least twice daily		2.0 (3.5)	2.4 (3.4)
	Less than twice daily		2.5 (3.5)	2.9 (3.6)

RDAS: Revised Dyadic Adjustment Scale; EPDS: Edinburgh Postnatal Depression Scale; SCL-90^ANX^: Symptom Checklist 90 Anxiety Subscale.

† *P* < 0.05 for chi-squared tests separately for each time point and within the groups of fathers.

# *P* < 0.05 for Mann Whitney U test within the groups of fathers.

‡ Missing values: negative and positive life events at 48 months: *n* = 91, RDAS at 48 months: *n* = 101. Others have less than 15 missing values.

Table 4 presents the results of the multivariable longitudinal regression analysis examining factors associated with mother-reported poor brushing of a child’s teeth during the study period. The brushing of a child’s teeth changed over time. If the mother brushed her teeth poorly, the likelihood of brushing of her child’s teeth poorly increased as the child grew older. However, the 95% confidence intervals for the mother’s toothbrushing were wide, indicating uncertainty. Also, if the father brushed his teeth poorly, the likelihood of mother-reported brushing of a child’s teeth poorly was greater.

**Table 4:** Multivariable longitudinal regression model for the mother-reported brushing of a child’s teeth less than twice daily (ref. brushing at least 2×day) as a function of time (12 mos, 24 mos, 48 mos), family-related variables and parents’ own toothbrushing (*n* = 305 children).

Categories		OR	95% CI	*P*
Time (ref. at 12 mos)	at 24 mos	0.37	0.25–0.54	< 0.001
	at 48 mos	0.22	0.13–0.39	< 0.001
Time × Mother’s own less than 2×day toothbrushing (ref. toothbrushing at least 2×day)	at 12 mos	6.92	3.04–15.51	< 0.001
	at 24 mos	9.91	4.64–20.36	< 0.001
	at 48 mos	29.18	10.02–84.39	< 0.001
Time × Mother’s low education (ref. medium/high)	at 12 mos	1.07	0.53–2.19	0.843
	at 24 mos	1.13	0.50–2.54	0.771
	at 48 mos	0.31	0.08–1.16	0.081
Father’s own less than 2×day toothbrushing (ref. toothbrushing at least 2×day)		1.77	1.04–3.03	0.037
One point change in RDAS		0.99	0.97–1.01	0.340
Father’s low education (ref. medium/high)		1.36	0.77–2.38	0.286
Mother’s age		1.07	0.98–1.14	0.144
Father’s age		1.02	0.97–1.09	0.410
Siblings (ref. No siblings)		1.12	0.74–1.69	0.591
At least one positive life event of father (ref. No positive life events father)		0.93	0.62–1.40	0.734

Mos: months; RDAS: Revised Dyadic Adjustment Scale.

Table 5 presents the results of similar regression analysis as in Table 4, but the dependent variable, child’s poor toothbrushing, was reported by father. Similar to mother’s model, the brushing of a child’s teeth changed over time. When the father or mother brushed their own teeth less than twice daily, the father was more likely to brush the child’s teeth less than twice daily. The main difference between these models was that the association between the mother’s own toothbrushing and the brushing of a child’s teeth varied across different time points, whereas the association with the father’s brushing remained consistent. Overall, both parents’ own toothbrushing was the strongest predictor in both models.

**Table 5:** Multivariable longitudinal regression model for the father-reported brushing of a child’s teeth less than twice daily (ref. brushing at least 2×day) as a function of time (12 mos, 24 mos, 48 mos), family-related variables and parents’ own toothbrushing (*n* = 307 children).

Categories		OR	95% CI	*P*
Time (ref. at 12 mos)	at 24 mos	0.53	0.39–0.72	< 0.001
	at 48 mos	0.32	0.21–0.47	< 0.001
Father’s own less than 2×day toothbrushing (ref. toothbrushing at least 2×day)		3.68	2.17–6.24	< 0.001
Mother’s own less than 2×day toothbrushing (ref. toothbrushing at least 2×day)		3.82	2.15–6.81	< 0.001
One point change in RDAS		1.02	0.99–1.04	0.464
Father’s low education (ref. medium/high)		1.04	0.62–1.75	0.883
Mother’s low education (ref. medium/high)		1.31	0.70–2.44	0.394
Mother’s age		1.05	0.98–1.13	0.264
Father’s age		1.02	0.96–1.08	0.393
Siblings (ref. No siblings)		1.08	0.75–1.56	0.721
At least one positive life event of father (ref. No positive life events father)		0.80	0.57–1.13	0.202

Mos: months; RDAS: Revised Dyadic Adjustment Scale.

## Discussion

Between the ages of 1 and 4 years, toothbrushing habits fluctuated in approximately one-third of the children, while two-thirds exhibited stable brushing habits. Half of the children had stable good toothbrushing habits and one-sixth stable poor brushing habits from 1 to 4 years. Both parents’ less than twice daily brushing was strongly associated with less than twice daily brushing of a child’s teeth from 1 to 4 years of age. When mother brushed her own teeth less than twice daily, the likelihood of brushing of her child’s teeth less than twice daily increased over time. In contrast, the association between the father’s less than twice daily brushing and the child’s brushing remained consistent throughout the study.

Few longitudinal and representative studies have examined preschool children’s toothbrushing and the association of both parents’ own toothbrushing with it, as in this study [5, 15, 16, 57, 58]. Notably, this study also included the children’s fathers, whose role in brushing a child’s teeth has received very limited research attention [6, 13, 15, 16, 22]. The large birth cohort sample has been shown to represent the general population in the region. However, in our study, the dropout rate was high due to the requirement that both parents answer at least one toothbrushing‑related question. This may limit the generalizability of the findings to all Finnish families with young children [46]. Families with more than one child and families with older and more highly educated parents were more likely to remain in the study and to be included in our study population.Hence, these groups were slightly overrepresented [59], and findings about toothbrushing frequencies of parents and their children might be better than they would be in the general population. Nevertheless, compared to the national study among women and men of a similar age in Finland, toothbrushing frequencies were similar among women but only slightly better among men [59, 60] in our study. Furthermore, no statistically significant differences in toothbrushing frequencies were found between mothers and fathers whose partners did not participate in the study and those whose partners did.

Another limitation of this study was using self-reported questionnaires when collecting data. Self-reported answers could lead to more socially acceptable answers. However, the good agreement between parents in reporting their child’s toothbrushing frequency at 4 years of age (κ = 0.80), together with findings from our previous studies [15, 16], supports the convergent validity of the parent-reported toothbrushing frequency data. Additionally, the number of fathers was lower than that of mothers, possibly due to the recruitment process during ultrasonography visits. As information on whether the mothers attended these visits alone or with the father/partner was not collected, we cannot calculate the percentage of the partners who were asked to participate. Among the mothers, 97.3% reported cohabiting with the child’s father or other male partner at the baseline. The data included only one two-female couple, thus limiting the findings to heterosexual couples. Finally, in our data, families did not experience many life events, symptoms of anxiety or depression, so the generalizability of these findings may be limited.

The finding that mother-reported brushing of a child’s teeth improved from age one to four, with the biggest improvement occurring between 1 and 2 years is not totally in line with previous studies. For instance, Wigen and Wang [6] reported the greatest increase in toothbrushing twice daily between the ages of three and five. Similarly, Rajesh et al. [22] found that toothbrushing twice daily increased notably from 2 to 5 years of age. In contrast, our findings indicate only a modest improvement in toothbrushing frequency after the age of two. Additionally, during the study period, toothbrushing frequency fluctuated in only one-third of the children, while two-thirds had stable brushing habits. Half of the children had stable good toothbrushing habits in our study. Among those whose teeth were brushed at least twice daily at age one, 86% maintained this habit until the age of four. Wigen and Wang reported similarly that among the children who had their teeth brushed twice daily at 1.5 years of age, the majority – more than four-fifths – continued this behavior at 3 years of age and at 5 years of age. These findings suggest that when toothbrushing is initiated around the age of one – due to the eruption of teeth – the behavior is often established by the age of two and appears to be relatively sustainable thereafter. Thus, for most children, brushing becomes a consistent habit from an early age.

The final multivariable longitudinal regression analysis indicated that both parents’ less than twice daily brushing was strongly associated with less than twice daily brushing of a child’s teeth from 1 to 4 years of age. Previous studies have reported strong cross-sectional associations between mother’s own poor toothbrushing and the poor brushing of her child’s teeth [8–13, 22, 59–63], but only a few studies have examined longitudinal associations between both father’s and mother’s toothbrushing and brushing of a child’s teeth [12–16, 22, 58, 64]. However, mother’s and father’s brushing behavior was examined often as a combined variable [13, 14, 22], not as a separate variable – as in our studies and in the study of Astrøm [15, 16, 64]. When studied simultaneously, the roles of the mother and father differ slightly. The mother’s role tends to be more prominent in the care of young children [15]. As the child grows, parents’ reporting on toothbrushing frequency of a child tends to become more consistent, as demonstrated in this study, and thus the father’s role probably becomes more pronounced. Additionally, the association between parent’s own toothbrushing and the brushing of a child’s teeth was different for mothers and fathers. Overall, there is limited research examining the father’s role in brushing his child’s teeth both cross-sectionally and longitudinally.

Parents’ level of education was not associated with the brushing frequency of a child from 1 to 4 years of age. This differs from our earlier study, in which lower parental education was associated with poor toothbrushing at age two, while other family-related variables were not [16]. However, the same family-related variables were not included in our previous analysis as in this study, which may partly explain the different findings. Partly in contrast to our findings, Rajesh et al. [22] reported that several sociodemographic factors (including mother’s educational level) were associated with toothbrushing frequency at 2 years of age, although only the child’s sex and mother’s toothbrushing remained significant at age five. In contrast, Wigen and Wang [6] found no association between good toothbrushing from 1.5 to 5 years of age and parent’s education in their multivariable logistic regression analysis. The differences in the findings observed may be attributable to population-level variations or differences in the variables used across the studies. It is important to acknowledge the high level of education of the Finnish population, as well as the comprehensive and free healthcare available to children. Nevertheless, our findings suggest that the parent’s own brushing habits are a more significant explanatory factor than educational level.

Oral health behavior is shaped by a variety of interconnected factors. This study showed that toothbrushing behavior appears to become established at a very early age and parent’s brushing habits play a central role in children’s toothbrushing frequency. These findings support the importance of early family-based oral health interventions, beginning already during pregnancy or when families are expecting a child. By targeting the entire family, these interventions may improve healthier brushing habits among parents, thereby reinforcing positive routines within the household and enabling good oral hygiene and health for a child [22, 65]. Rather than relying solely on the provision of oral health information, interventions should actively engage parents in discussions about their oral health and daily routines. Evidence suggests that motivational interviewing may be more effective than traditional oral health education in improving parents’ oral health knowledge, attitudes, and behaviors, highlighting the importance of collaborative approaches to oral health promotion [23, 66]. Special attention should be given to families who experience difficulties in maintaining routines.

## Data Availability

Data may be shared as part of research collaboration in accordance with Finnish and EU legislation related to personal data protection and ethical issues in medical research. Access to data may be inquired by contacting the PI, Prof. Hasse Karlsson (hasse.karlsson@utu.fi) and co-PI Linnea Karlsson (linnea.karlsson@utu.fi).
